# Supplementary material for: Interplay between DNA damage repair and apoptosis shapes cancer evolution through aneuploidy and microsatellite instability
Source: Nat Commun. 2020 Mar 6;11:1234. doi: 10.1038/s41467-020-15094-2 (PMC7060240; doi:10.1038/s41467-020-15094-2)
Supplement: Supplementary file 1 — Supplementary Information [file 41467_2020_15094_MOESM1_ESM.pdf]

## SUPPLEMENTAL INFORMATION

# Interplay between DNA damage repair and apoptosis shapes cancer evolution through aneuploidy and microsatellite instability

**Auslander et al.**

## Supplementary Figure 1

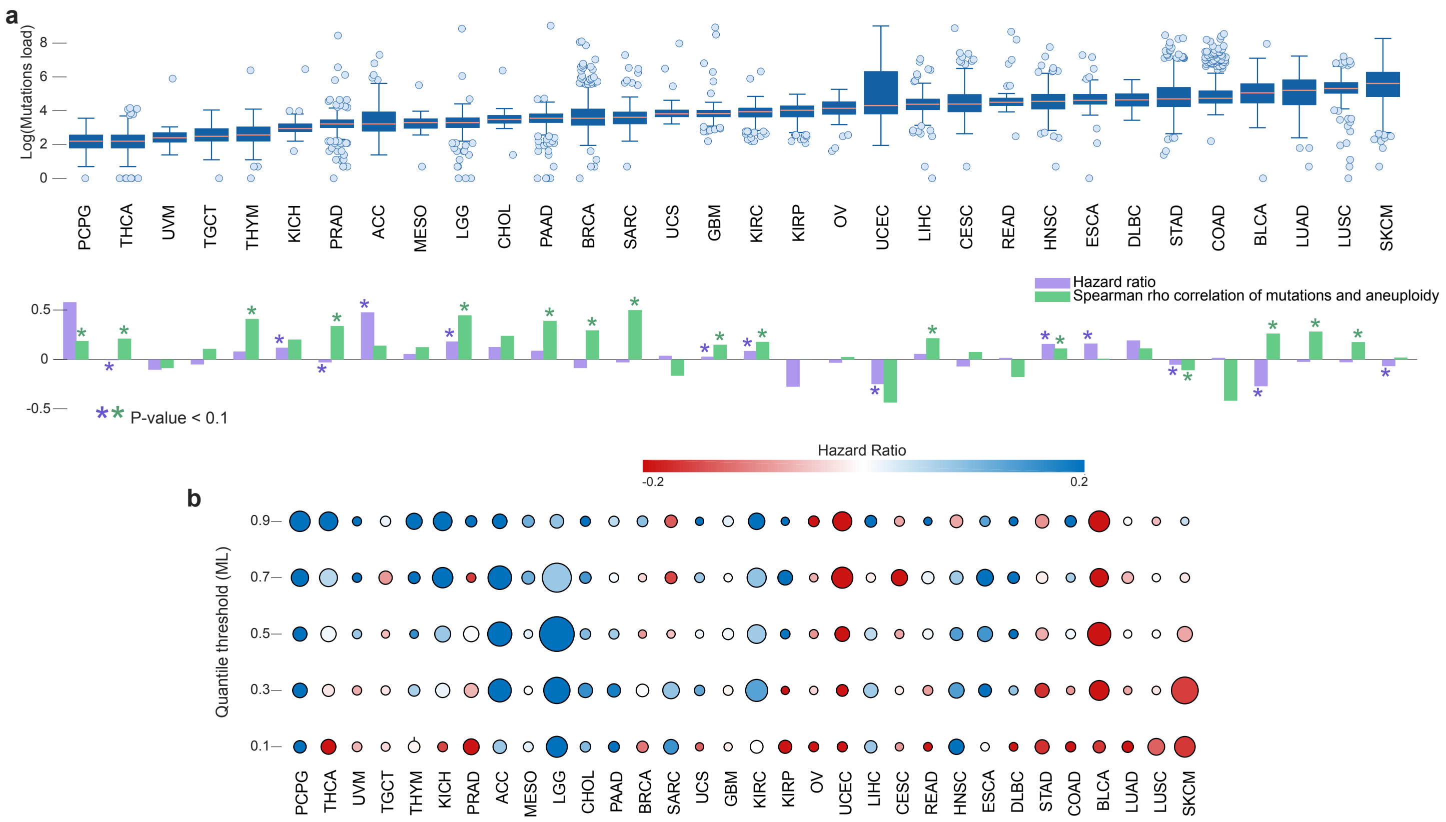


**Supplementary Figure 1** Pan-cancer association between the overall mutational load, levels of aneuploidy and overall survival.
**(a)** Top panel: Boxplots showing the distribution of the log of mutational load per sample in each tumor type. Center lines indicate medians, box edges represent the interquartile range, whiskers extend to the most extreme data points not considered outliers, and the outliers are plotted individually. Bottom panel: the corresponding correlation coefficients between the mutational load and aneuploidy scores, and the hazard ratio values resulting from Kaplan-Meier overall survival curves for samples with high vs. low number of driver mutations (separated by the median). Positive hazard ratio values indicate that high mutation load is associated with worse survival, and negative hazard ratio values indicate that high mutation load is associated with improved survival. Statistical significance (log-rank and Spearman rank-correlation P-value < 0.05) is indicated with *. **(b)** The hazard ratio values resulting from Kaplan-Meier overall survival prediction curves for samples with high vs. low number of driver mutations for different thresholds (y-axis), for different tumor types (x-axis). The circle sizes represent the significance level measured as log-rank P-value.

## Supplementary Figure 2

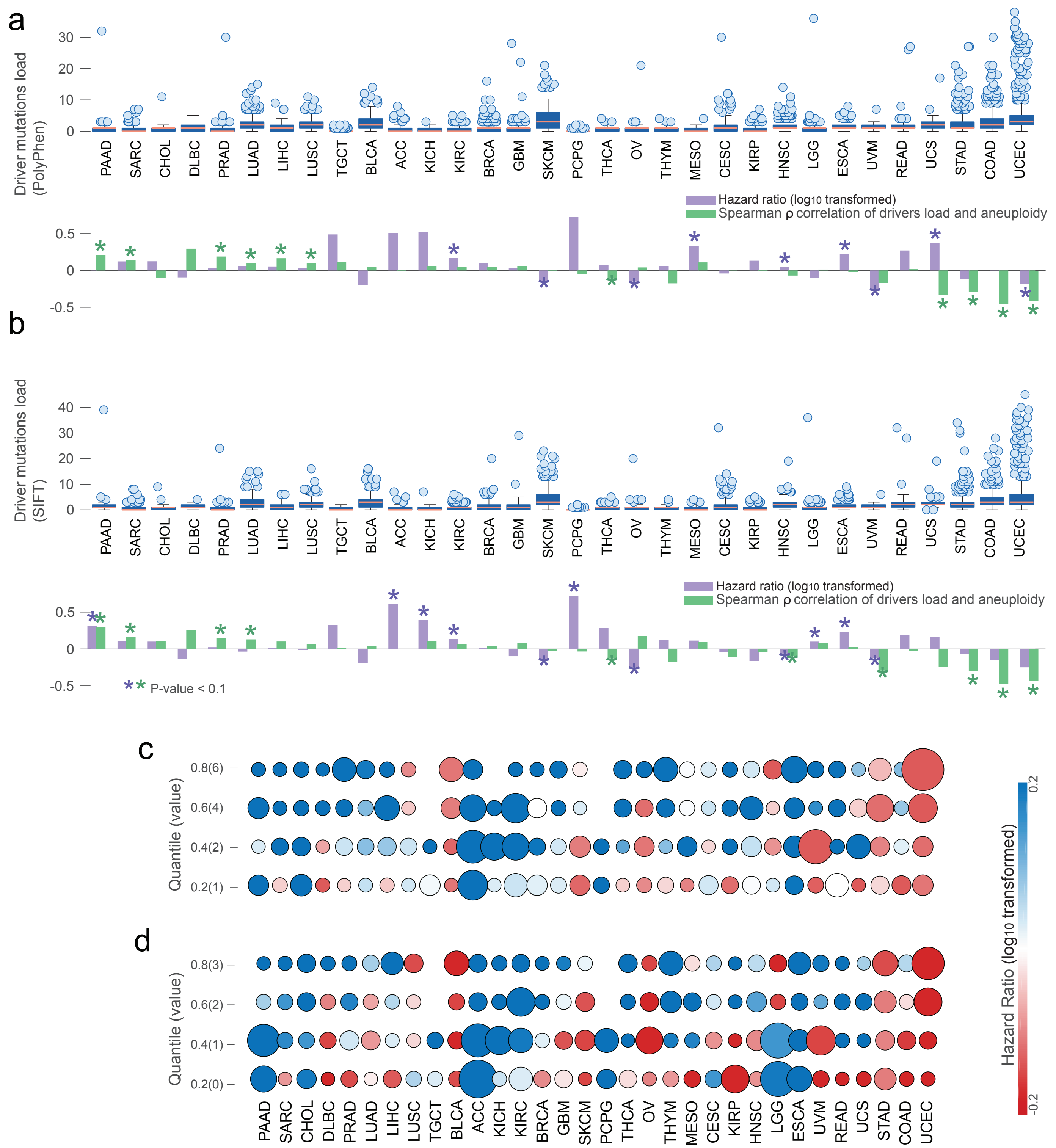


**Supplementary Figure 2** Pan-cancer association between the number of driver mutations (using SIFT and PolyPhen scores to identify drivers), levels of aneuploidy and overall survival.
**(a)-(b)** are equivalent to Figure 1a, but when defining the driver mutations load using only driver mutations with damaging PolyPhen scores **(a)** and when defining the driver mutations load using only driver mutations with deleterious SIFT scores **(b)**. **(c)-(d)** are equivalent to Figure 1b, but when defining the driver mutations load using only driver mutations with damaging PolyPhen scores **(c)** and when defining the driver mutations

load using only driver mutations with deleterious SIFT scores **(d)** (SIFT and PolyPhen scores are obtained from GCD MAF Format v.1.0.0.0)

**Supplementary Figure 3**

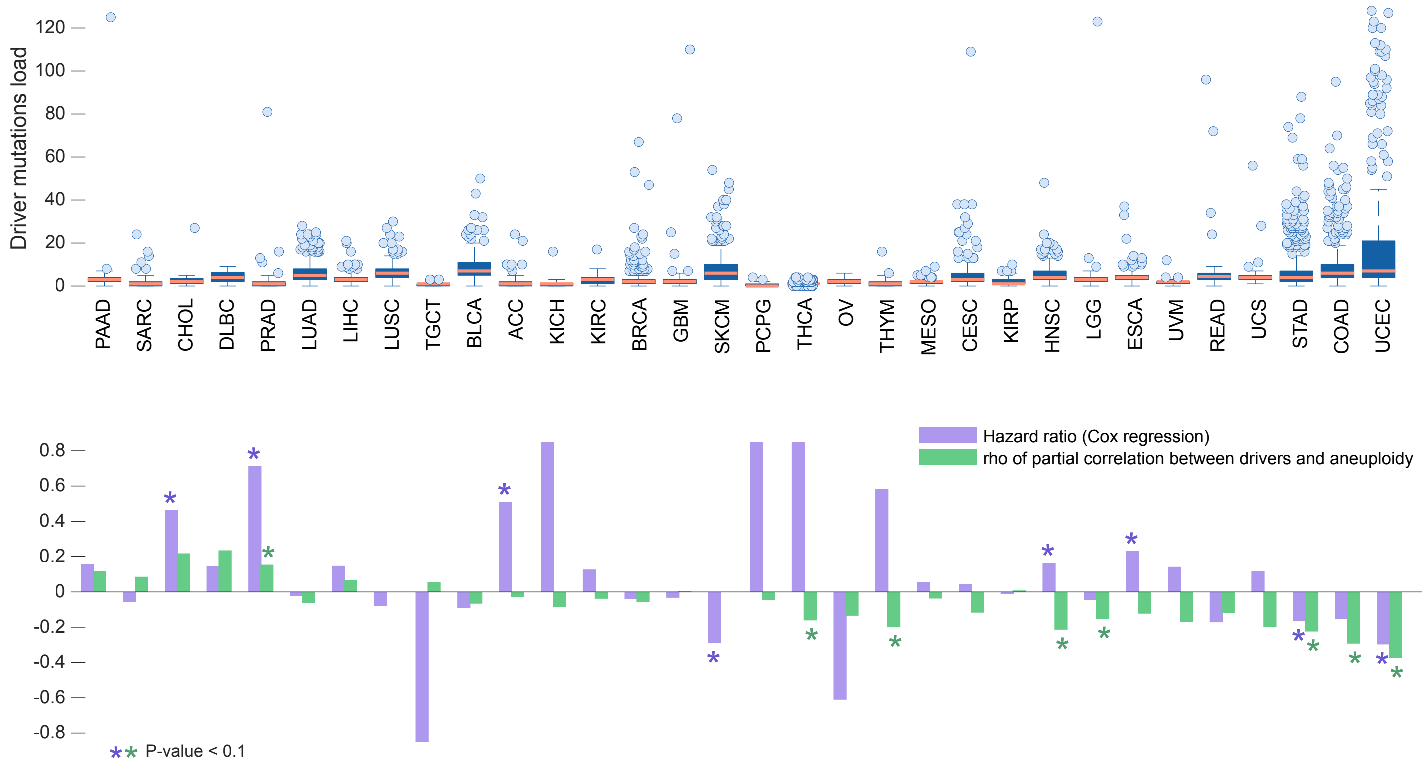


**Supplementary Figure 3** Pan-cancer association between the number of driver mutations, levels of aneuploidy and overall survival, controlling for overall mutational load as a continuous variable.
Top panel: distribution of the number of driver mutations per sample in each tumor type. Bottom panel: the corresponding partial correlation coefficients (controlling for total mutational burden) between the number of driver mutations and aneuploidy scores, and the hazard ratio values (log10 transformed) resulting from Cox-regression based on the number of driver mutations. Positive log10-hazard ratio values indicate that high load of driver mutations is associated with worse survival, and negative log10-hazard ratio values indicate that high load of drivers is associated with improved survival. Statistical significance (cox-regression and Spearman rank-correlation P-value < 0.05) is indicated with *.

**Supplementary Figure 4**

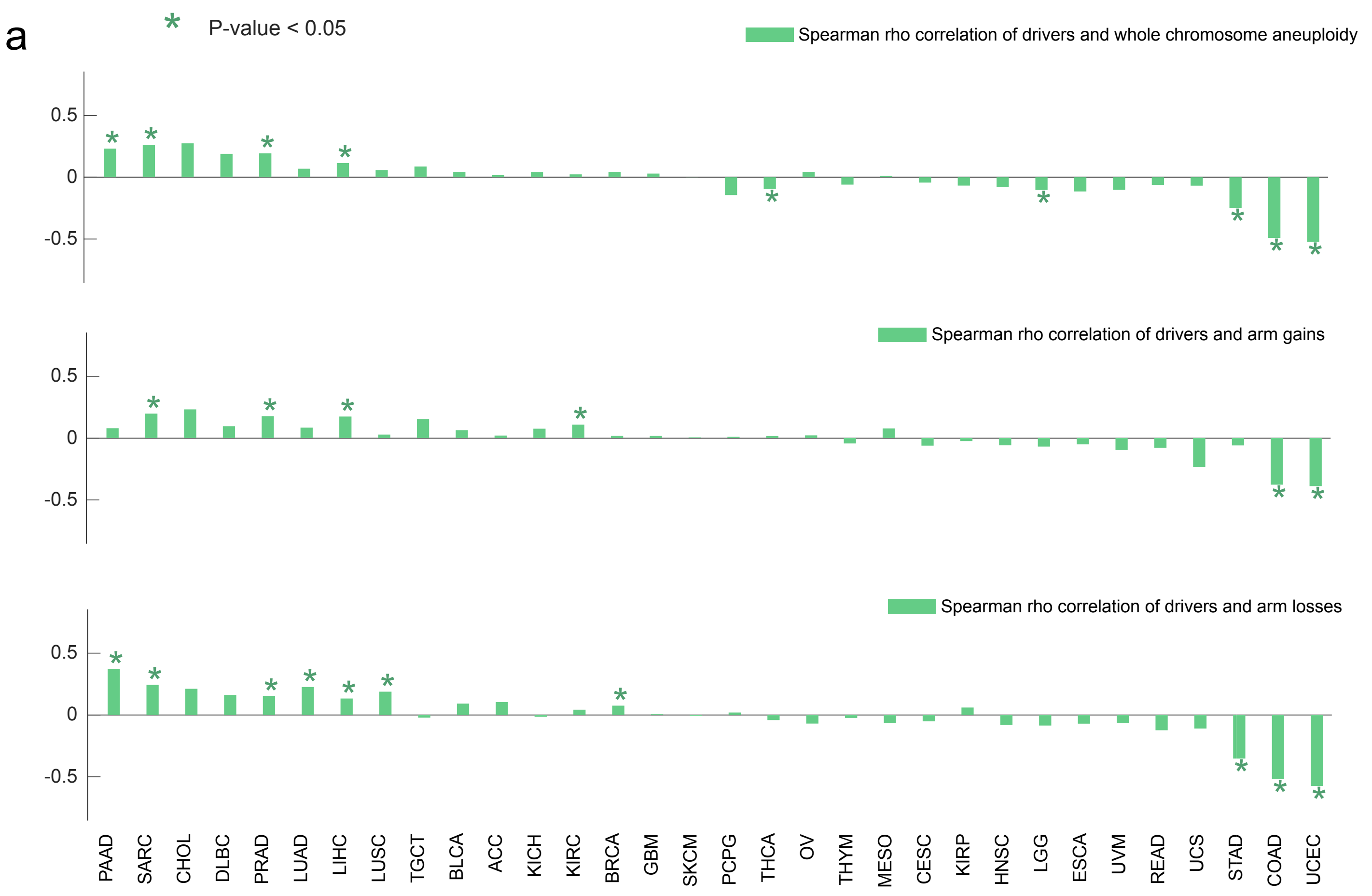


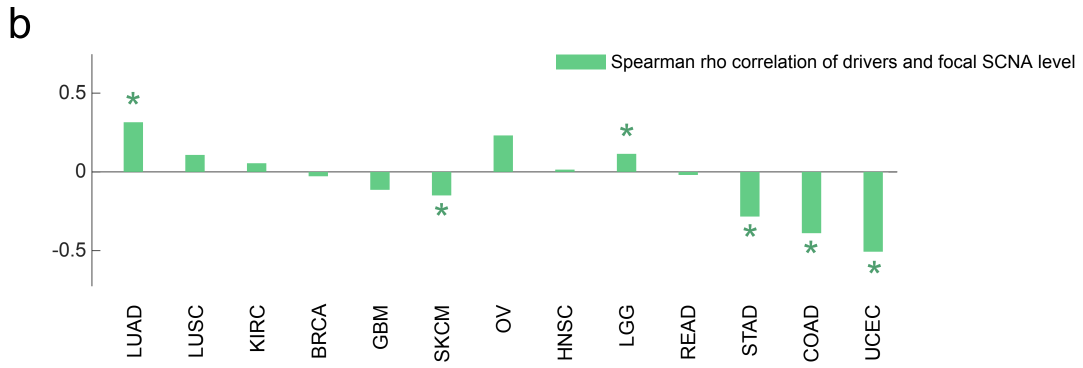


**Supplementary Figure 4** Pan-cancer association between the number of driver mutations, whole chromosome aneuploidy and arm gains and losses.

**(a)** The correlation coefficients between the number of driver mutations and aneuploidy, top panel: whole-chromosome aneuploidy scores , middle panel: arm levels gains and bottom panel: arm-level losses. Statistical significance (Spearman rank-correlation P-value < 0.05) is indicated with *. **(b)** the corresponding correlation coefficients between the number of driver mutations and the total count of SCNAs for tumor types with available SCNA count data. Statistical significance (Spearman rank-correlation P-value < 0.05) is indicated with *.

Supplementary Figure 5

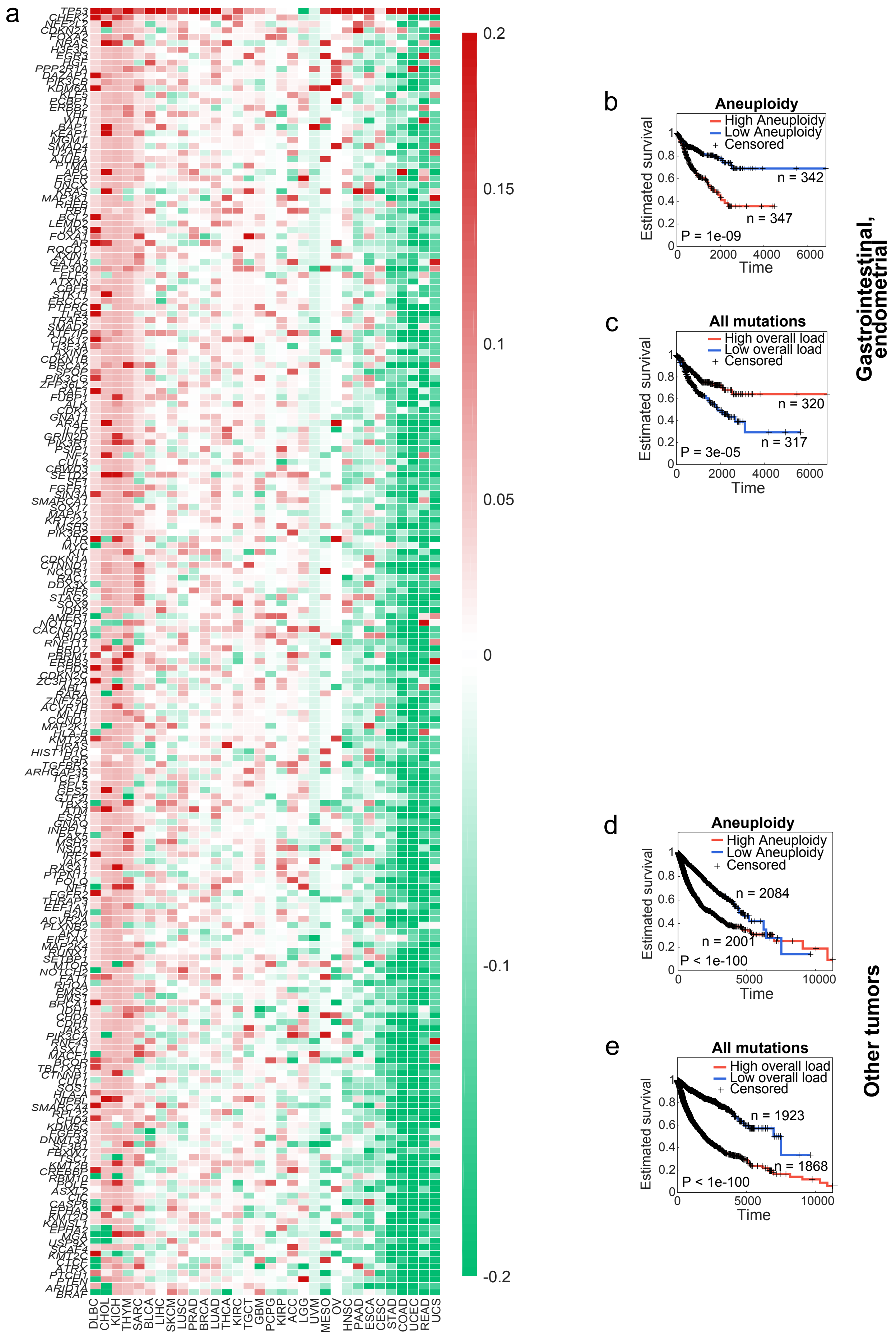

**Supplementary Figure 5**

Association map between aneuploidy and driver mutations, and survival correlates of overall mutational load and aneuploidy.

**(a)** Map of associations between aneuploidy and driver mutations (rows) for each tumor type (columns). **(b)** Kaplan Meier curves predicting overall survival for gastrointestinal and endometrial tumors with high vs. low aneuploidy levels, separated with the median. **(c)** Kaplan Meier curves predicting overall survival for gastrointestinal and endometrial tumors with high vs. low overall mutational load, separated with the median. **(d)** Kaplan Meier curves predicting overall survival for all other tumor types, with high vs. low aneuploidy levels, separated with the median. **(e)** Kaplan Meier curves predicting overall survival for all other tumor types, with high vs. low overall mutational load, separated with the median. The log-rank P-values are indicated for Kaplan Meier plots.

**Supplementary Figure 6**

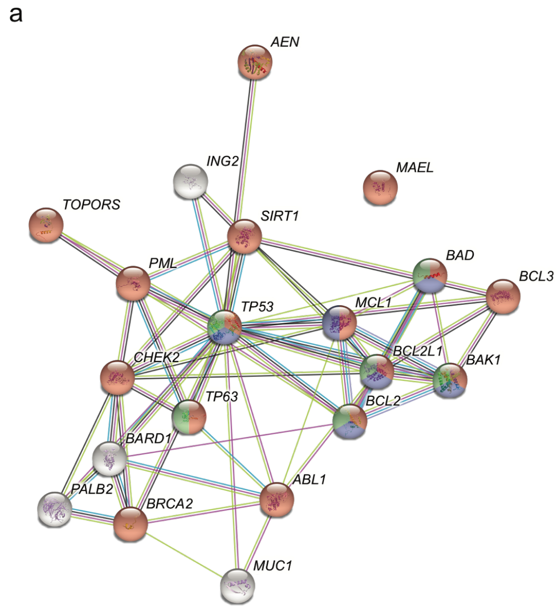


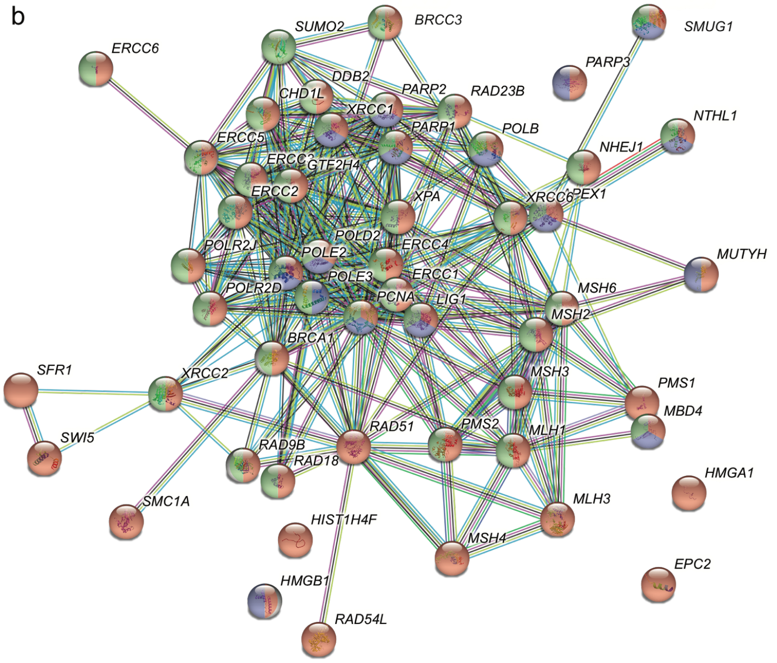


**Biological Process (GO)**

| GO-term | description | count in gene set | false discovery rate |
|---|---|---|---|
| GO:0008630 | intrinsic apoptotic signaling pathway in response to DNA d... | 16 of 71 | 5.39e-32 |
| GO:0006974 | cellular response to DNA damage stimulus | 19 of 749 | 2.07e-23 |
| GO:0042771 | intrinsic apoptotic signaling pathway in response to DNA d... | 9 of 30 | 1.09e-17 |
| GO:0042981 | regulation of apoptotic process | 18 of 1501 | 4.64e-16 |
| GO:0072331 | signal transduction by p53 class mediator | 10 of 128 | 5.71e-15 |

**KEGG Pathways**

| pathway | description | count in gene set | false discovery rate |
|---|---|---|---|
| hsa05206 | MicroRNAs in cancer | 7 of 149 | 1.11e-08 |
| hsa04210 | Apoptosis | 6 of 135 | 1.96e-07 |
| hsa05220 | Chronic myeloid leukemia | 5 of 76 | 3.13e-07 |
| hsa05212 | Pancreatic cancer | 5 of 74 | 3.13e-07 |
| hsa01524 | Platinum drug resistance | 5 of 70 | 3.13e-07 |

**Reactome Pathways**

| pathway | description | count in gene set | false discovery rate |
|---|---|---|---|
| HSA-109606 | Intrinsic Pathway for Apoptosis | 6 of 43 | 1.14e-09 |
| HSA-5693532 | DNA Double-Strand Break Repair | 6 of 147 | 6.16e-07 |
| HSA-5633007 | Regulation of TP53 Activity | 6 of 159 | 6.46e-07 |
| HSA-114452 | Activation of BH3-only proteins | 4 of 29 | 8.94e-07 |
| HSA-6785807 | Interleukin-4 and Interleukin-13 signaling | 5 of 106 | 1.87e-06 |

**Biological Process (GO)**

| GO-term | description | count in gene set | false discovery rate |
|---|---|---|---|
| GO:0006281 | DNA repair | 51 of 491 | 6.73e-75 |
| GO:0006259 | DNA metabolic process | 52 of 773 | 2.45e-68 |
| GO:0006289 | nucleotide-excision repair | 19 of 110 | 1.22e-26 |
| GO:0006310 | DNA recombination | 21 of 202 | 1.70e-25 |
| GO:0009314 | response to radiation | 25 of 425 | 3.22e-25 |

**KEGG Pathways**

| pathway | description | count in gene set | false discovery rate |
|---|---|---|---|
| hsa03410 | Base excision repair | 16 of 33 | 1.00e-28 |
| hsa03420 | Nucleotide excision repair | 15 of 46 | 5.32e-25 |
| hsa03430 | Mismatch repair | 9 of 23 | 1.64e-15 |
| hsa01524 | Platinum drug resistance | 7 of 70 | 1.77e-08 |
| hsa03030 | DNA replication | 5 of 36 | 8.09e-07 |

**Reactome Pathways**

| pathway | description | count in gene set | false discovery rate |
|---|---|---|---|
| HSA-73894 | DNA Repair | 39 of 290 | 2.98e-56 |
| HSA-5696398 | Nucleotide Excision Repair | 22 of 109 | 1.14e-32 |
| HSA-5696399 | Global Genome Nucleotide Excision Repair (GG-NER) | 19 of 83 | 6.26e-29 |
| HSA-5696400 | Dual Incision in GG-NER | 15 of 41 | 3.38e-25 |
| HSA-6781827 | Transcription-Coupled Nucleotide Excision Repair (TC-NER) | 16 of 78 | 1.39e-23 |

**Supplementary Figure 6** STRING network visualization and pathway enrichment of the apoptosis and repair gene sets.

**(a)** STRING network visualization and top pathways enriched (with GO, KEGG and REACTOME) for the apoptosis gene set.

**(b)** STRING network visualization and top pathways enriched (with GO, KEGG and REACTOME) for the repair gene set.

## Supplementary Figure 7

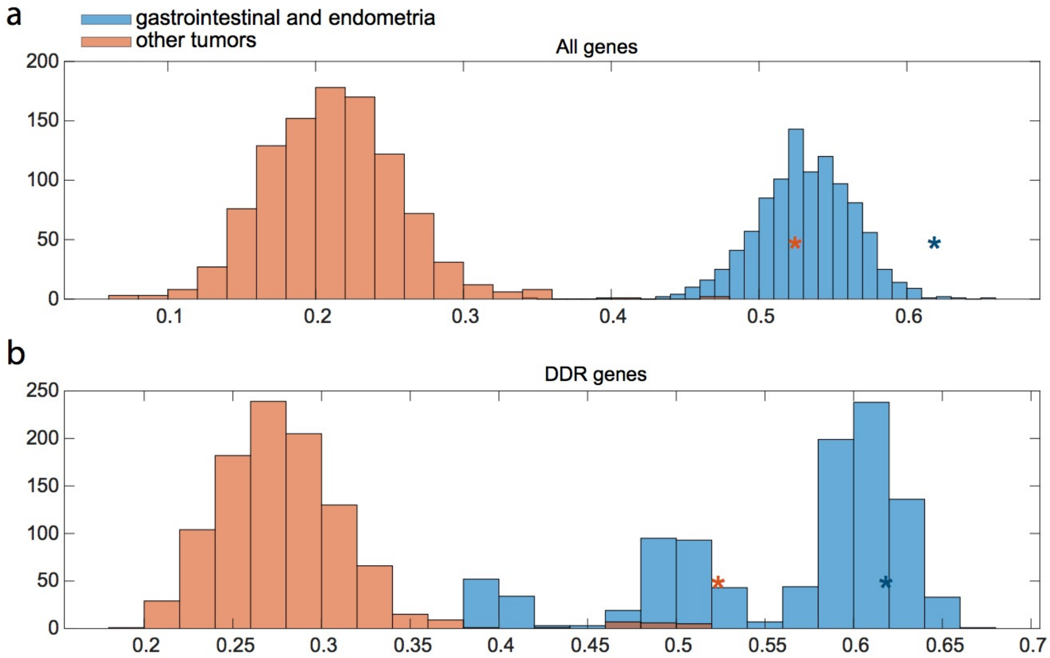


**Supplementary Figure 7** Correlations of randomly selected gene sets.
**(a)** histograms showing the number of randomly selected gene sets of mutations (y-axis, of total of 1000 random gene sets of mutations from all genes) for each level of correlation with the load of driver mutations (x-axis), in the gastrointestinal and endometrial tumors (blue) and all other tumors (orange). The correlation of the repair gene set is marked in blue *, and that of the apoptosis gene set is marked in orange *. **(b)** histograms showing the number of randomly selected gene sets of mutations (y-axis, of total of 1000 random gene sets of mutations from DDR genes) for each level of correlation with the load of driver mutations (x-axis), in the gastrointestinal and endometrial tumors (blue) and all other tumors (orange). The correlation of the repair gene set is marked in blue *, and that of the apoptosis gene set is marked in orange *.

## Supplementary Figure 8

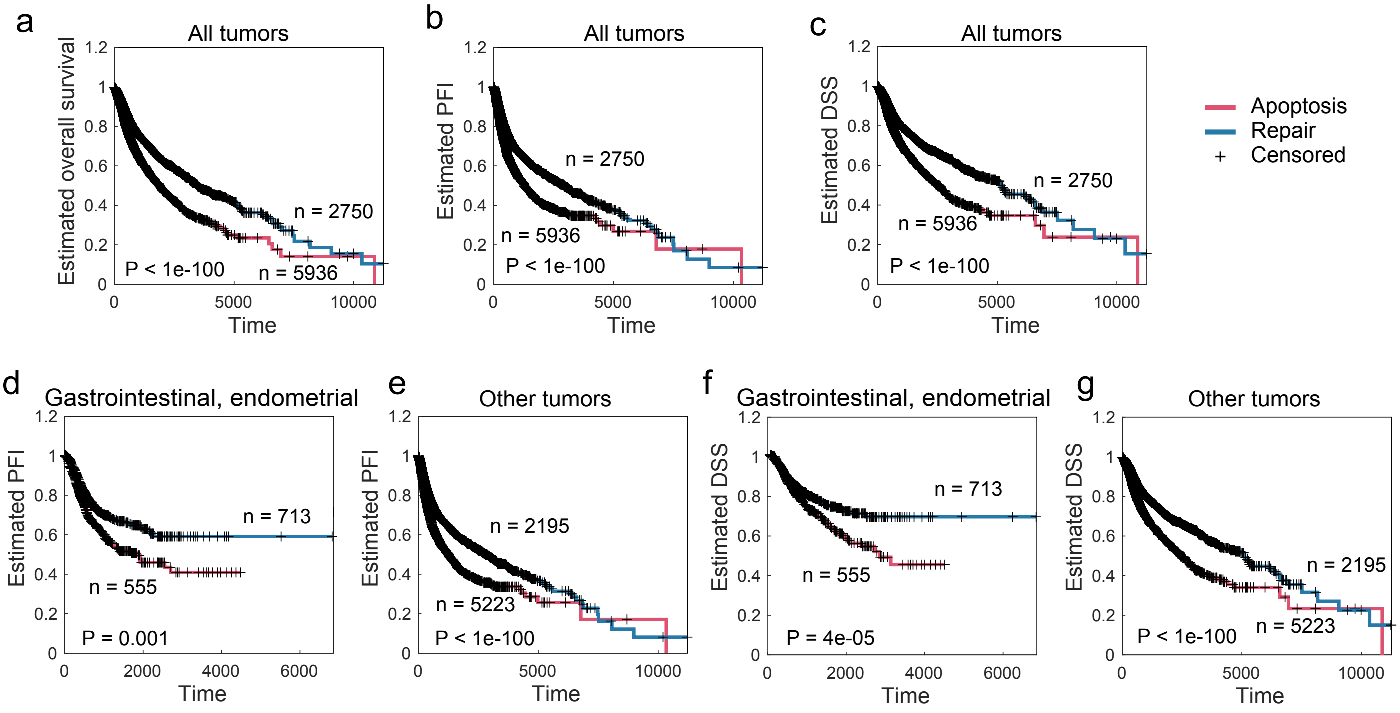

**Supplementary Figure 8** Repair and apoptosis sets correlates of clinical outcome.
Kaplan Meier curves predicting **(a)** overall survival, **(b)** Progression free interval (PFI) and **(c)** disease specific survival (DSS), for patients with higher repair set mutation rate (i.e. more repair set mutations than apoptosis set mutations, blue) vs. those with higher apoptosis set mutation rate (red), for all tumors, and predicting PFI **(d),(e)** and DSS **(f),(g)** separately for gastrointestinal and endometrial tumor samples, and all other tumor samples. The log-rank P-values are indicated.

## Supplementary Figure 9

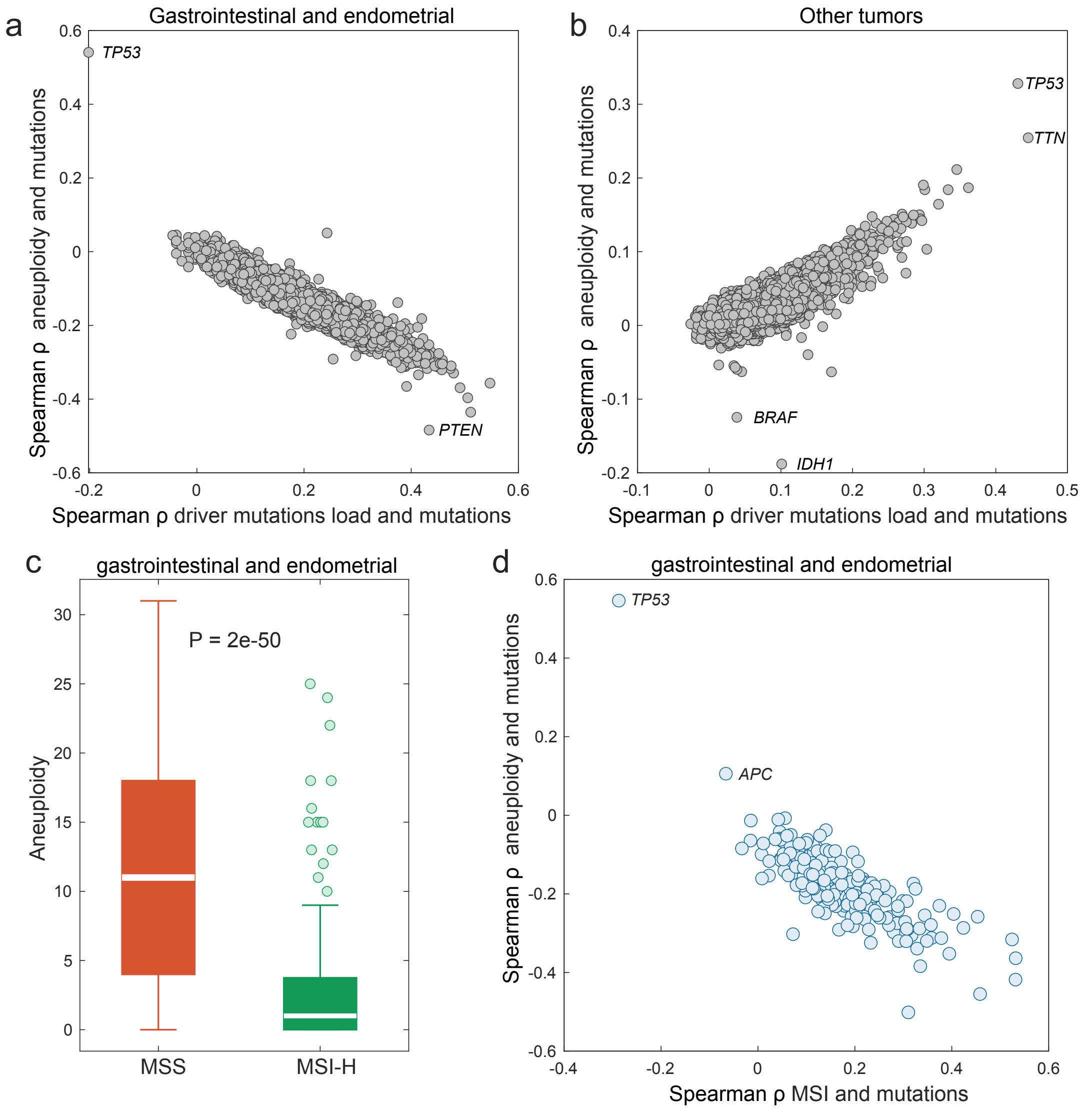


**Supplementary Figure 9** Driver mutations correlates of aneuploidy, driver mutations load and MSI.
**(a)** The correlation coefficient between the occurrence of mutations in each gene and the load of driver mutations (x-axis) and the correlation coefficient between the occurrence of mutations in each gene and aneuploidy (y-axis), in gastrointestinal and endometrial tumors. **(b)** The correlation coefficient between the occurrence of mutations in each gene and the load of driver mutations (x-axis) and the correlation coefficient between the occurrence of mutations in each gene and aneuploidy (y-axis), in all other tumors. **(c)** Aneuploidy levels in MSS (orange) vs. MSI-H (green) tumors. The rank-sum P-value is indicated. **(d)** The correlation coefficient between the occurrence of mutations in each gene and MSI (x-axis) and the correlation coefficient

between the occurrence of mutations in each gene and aneuploidy (y-axis). TP53 and APC are marked, as outliers with different patters compared with other driver genes.

## Supplementary Figure 10

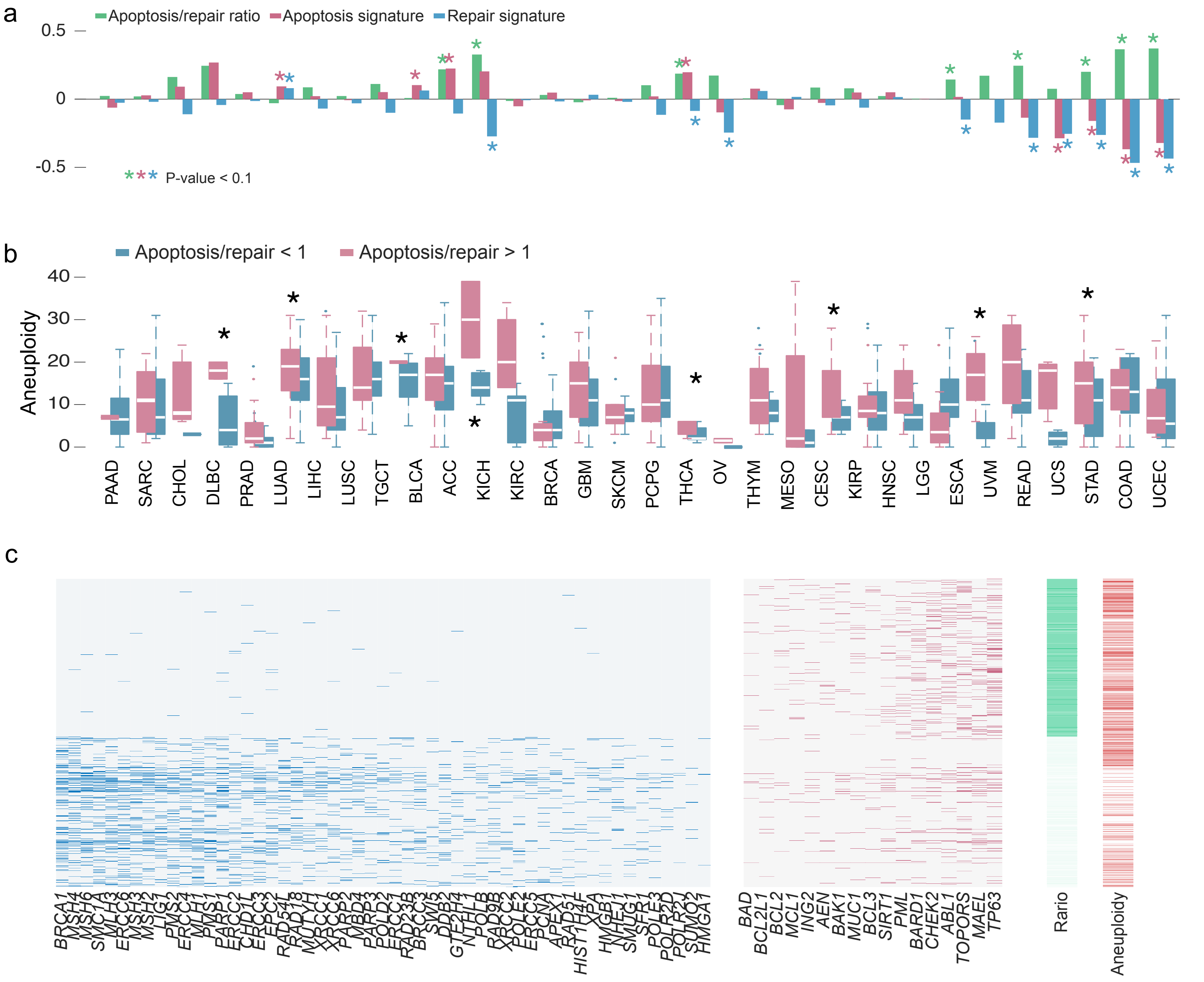


**Supplementary Figure 10** DNA repair and apoptosis (excluding TP53 and BRCA2) mutational sets correlate with aneuploidy.
**(a)** Spearman correlation coefficient between aneuploidy and the apoptosis (when excluding TP53 and BRCA2) and repair set load, and their ratio. Statistical significance (Spearman rank-correlation P-value < 0.1) is indicated with *. **(b)** Boxplots comparing the aneuploidy distribution between low (<1) and high (>1) apoptosis (when excluding TP53 and BRCA2) to repair set load ratio. Statistical significance (Rank-sum P-value < 0.1) is indicated with *. Center lines indicate medians, box edges represent the interquartile range, whiskers extend to the most extreme data points not considered outliers, and the outliers are plotted individually. **(c)** Map of the repair and apoptosis (when excluding TP53 and BRCA2) set mutations and levels of aneuploidy, for samples with highest and lowest apoptosis to repair set load ratios (top and bottom 0.05 quartiles).

## Supplementary Figure 11

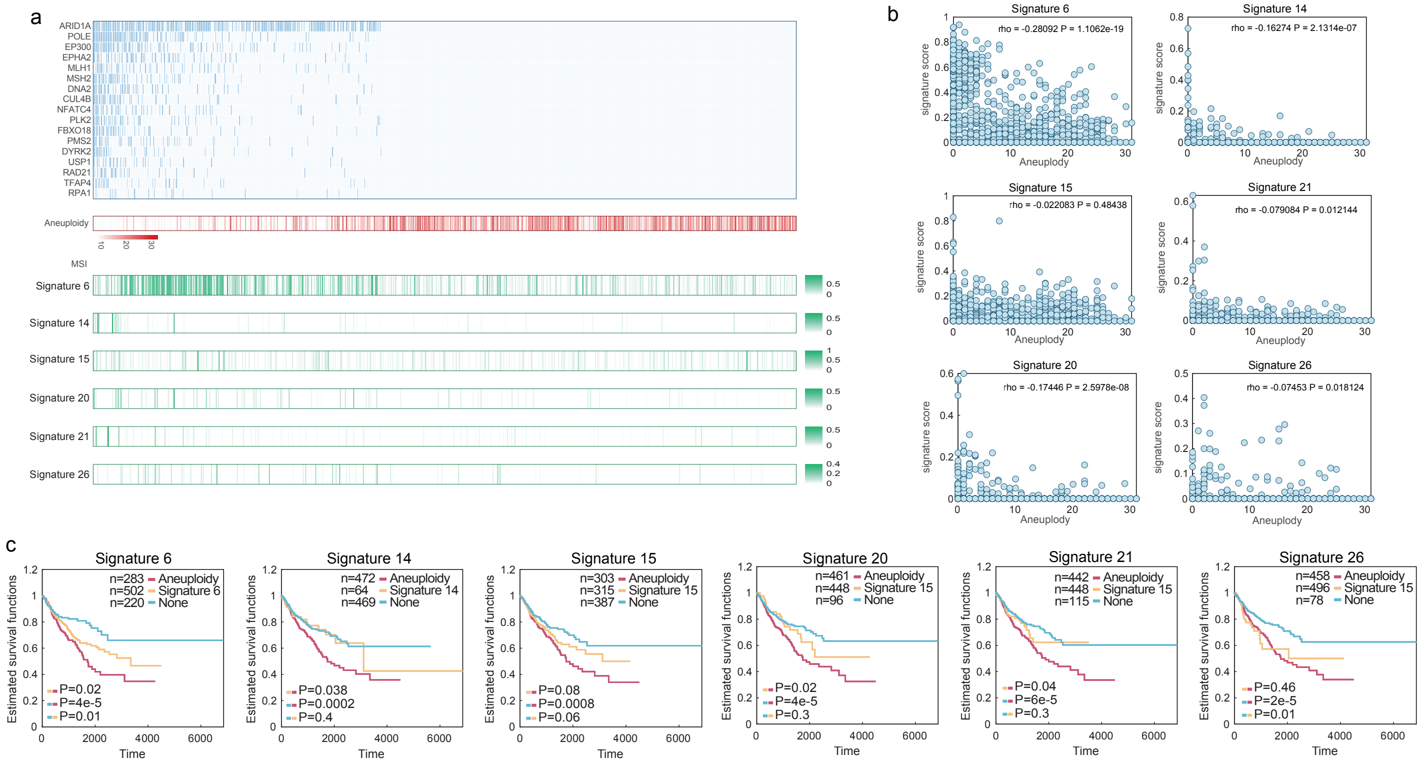


**Supplementary Figure 11** COSMIC MSI associated signatures correlates with aneuploidy and overall survival rates.

**(a)** The mutations in the selected MSI-aneuploidy set (top panel) negatively correlate with aneuploidy (middle panel) and positively correlate with MSI mutational signatures scores (bottom panels) across gastrointestinal and endometrial tumor samples (COSMIC signatures 6, 14, 15, 20, 21 and 26, that has been associated with MSI**). (b)** correlations between aneuploidy (x-axes) and MSI mutational signatures (y-axes). **(c)** Kaplan Meier overall survival curves for tumors with high aneuploidy (higher than median) and low MSI mutational signatures scores (lower than median, red curve), tumors with low aneuploidy (lower than median) and high MSI mutational signatures scores (higher than median , yellow curve) and tumors with low MSI mutational signatures scores (lower than median) and low aneuploidy (blue curve, lower than median). The log-rank P-value are provided.

## Supplementary Figure 12

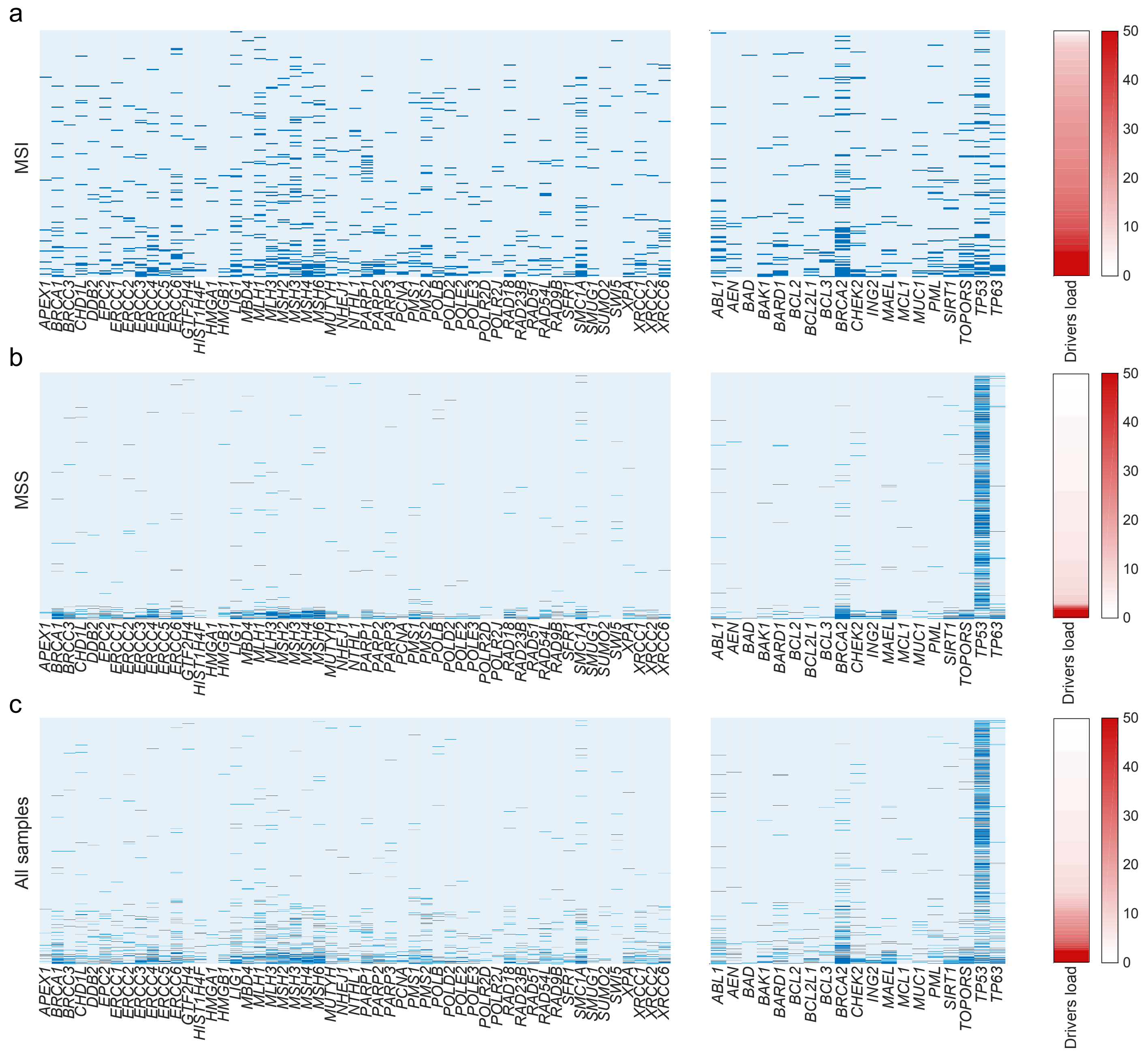


**Supplementary Figure 12** The repair and apoptosis gene sets correlate with driver mutations load specifically in MSS and MSI tumors.

Mutations in the repair (left panels) and apoptosis (middle panels) gene sets vs the load of driver mutations (right panels), for MSI tumors **(a)**, MSS tumors **(b)** and for all tumors **(c)**.

## Supplementary Figure 13

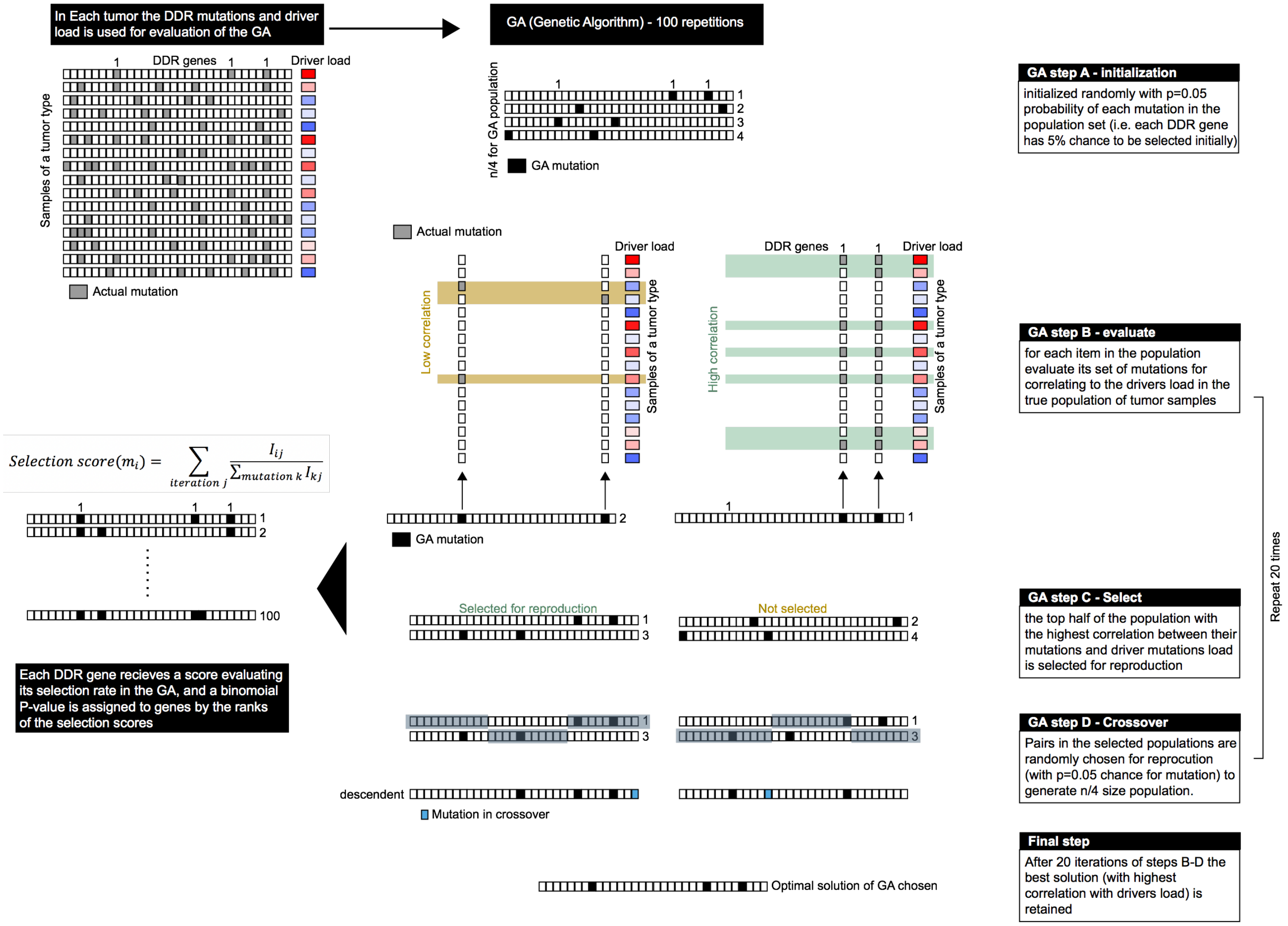


**Supplementary Figure 13** Schematic outline of the process employed to select DDR gene sets that correlate with driver mutations load (employed for the selection of the apoptosis and repair gene sets).

## Supplementary Figure 14

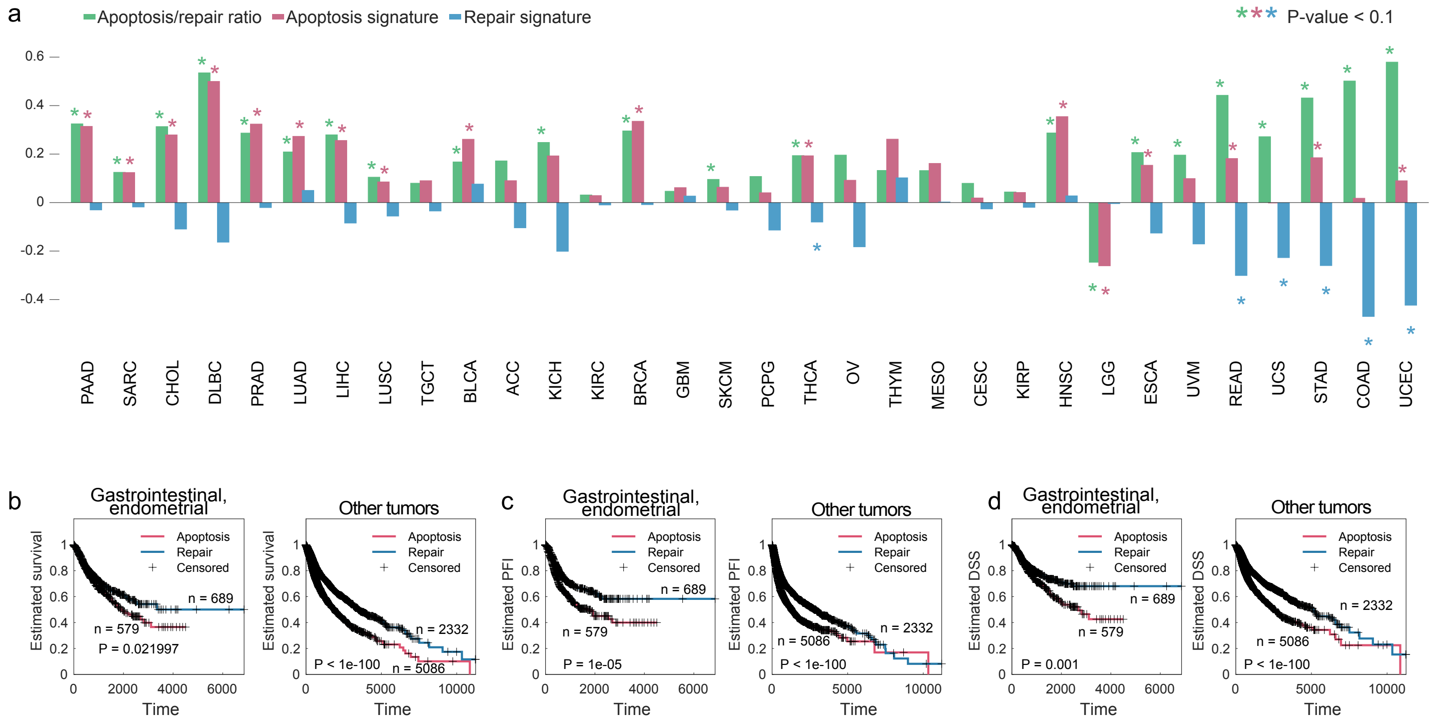


**Supplementary Figure 14** Repair and apoptosis gene sets derived using $X^2$ P-value<0.05
**(a)** Spearman correlation coefficient between aneuploidy and the apoptosis and repair set load, and their ratio. Statistical significance (Spearman rank-correlation P-value < 0.1) is indicated with *. **(b)** Kaplan Meier curves predicting overall survival for patients with higher repair set mutation rate (i.e. more repair set mutations than apoptosis set mutations, blue) vs. those with higher apoptosis set mutation rate (red), for gastrointestinal and endometrial tumor samples (left panel) and all other tumor samples (right panel).**(c)** Kaplan Meier curves predicting progression free interval (PFI) for patients with higher repair set mutation rate (blue) vs. those with higher apoptosis set mutation rate (red), for gastrointestinal and endometrial tumor samples (left panel) and all other tumor samples (right panel). **(d)** Kaplan Meier curves predicting disease specific survival (DSS) for patients with higher repair set mutation rate (blue) vs. those with higher apoptosis set mutation rate (red), for gastrointestinal and endometrial tumor samples (left panel) and all other tumor samples (right panel).

## Supplementary Figure 15

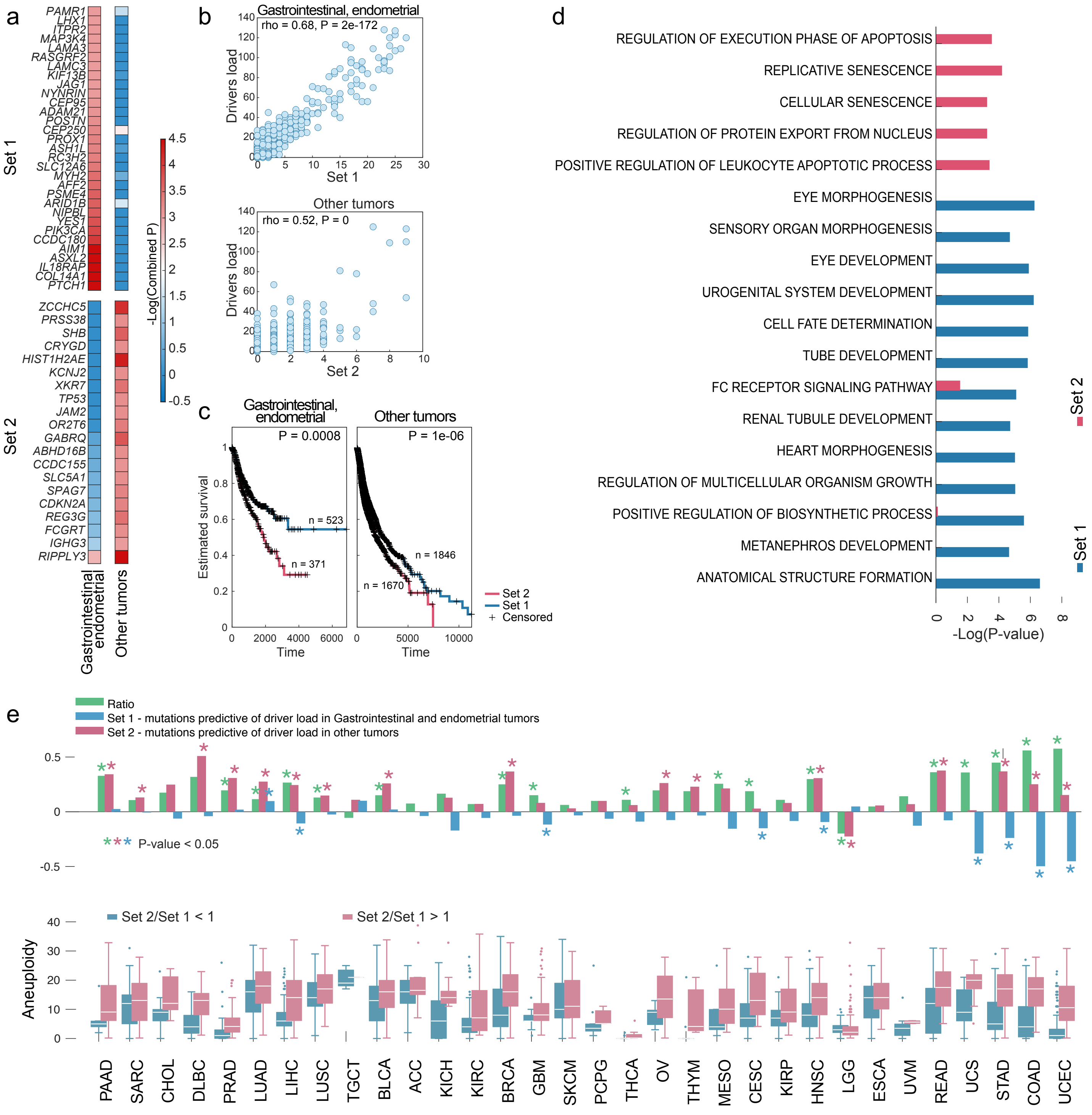

**Supplementary Figure 15** The gene sets derived starting with all genes (not limited to DDR genes) **(a)** The combined selection P-values for each cluster of tumors, for each selected gene (rows). **(b)** scatter plots correlating the driver mutation load (y-axes) with the gene set 1 load (upper panel x-axis, for gastrointestinal and endometrial tumors), and with  the gene set2 load (bottom panel x-axis, for all other tumor types). **(c)** Kaplan Meier curves predicting overall survival for patients with higher set1 mutation rate  vs. those with higher set2 mutation rate (red), for gastrointestinal and endometrial tumor samples (left panel) and all other tumor samples (right panel) **(d)** hyper-geometric enrichment P-value for pathways enriched with genes from one of the selected sets. **(e)** top panel: Spearman correlation coefficient between aneuploidy and the two gene sets load, and their ratio. Statistical significance (Spearman rank-correlation P-value < 0.05) is indicated with *.

Bottom panel: Boxplot comparing the aneuploidy distribution between low (<1) and high (>1) set1 to set2 load ratio. Statistical significance (Rank-sum P-value < 0.05) is indicated with *. Center lines indicate medians, box edges represent the interquartile range, whiskers extend to the most extreme data points not considered outliers, and the outliers are plotted individually.
