# Supplementary material for: Interplay between DNA damage repair and apoptosis shapes cancer evolution through aneuploidy and microsatellite instability
Source: Nat Commun. 2020 Mar 6;11:1234. doi: 10.1038/s41467-020-15094-2 (PMC7060240; doi:10.1038/s41467-020-15094-2)
Supplement: Supplementary file 3 — Description of Additional Supplementary Files [file 41467_2020_15094_MOESM3_ESM.pdf]

Description of Additional Supplementary Files

File Name: Supplementary Data 1
Description: Aneuploidy and clinical data for 8686 TCGA tumor samples considered

File Name: Supplementary Data 2
Description: Gene grouping into DNA damage response pathways

File Name: Supplementary Data 3
Description: Pan cancer driver genes considered

File Name: Supplementary Data 4
Description: Selection p-values assigned to selected DNA damage response genes in each tumor type (binomial p-value) and tumor cluster (X^2 P-values)
